# Pharmacogenomics of CRC treatments: putting bricks on the path to personalized medicine

**DOI:** 10.1186/1471-2164-15-S2-O5

**Published:** 2014-04-02

**Authors:** Ceres Fernández-Rozadilla, Emilia Balboa, Mahmood Rasool, Francisco Barros, Antoni Castells, Sergi Castellvi-Bel, Alejandro Brea-Fernández, Clara Ruiz-Ponte, Angel Carracedo

**Affiliations:** 1 Genomic Medicine Group-CIBERER. University of Santiago de Compostela, 15782 Santiago de Compostela, Spain; 2 Molecular and Population Genetics Laboratory, Wellcome Trust Centre for Human Genetics, Roosevelt Drive, Oxford OX3 7BN, UK; 3 Center of Excellence in Genomic Medicine Research. King Abdulaziz University, Post Box: 80216 Jeddah 21589, KSA; 4 Servicio de Gastroenterología, Hospital Clínic, IDIBAPS, Universitat de Barcelona, CIBERehd, Barcelona, Spain; 5 Galician Foundation of Genomic Medicine, IDIS, SERGAS, 15706 Santiago de Compostela, Spain

## 

Colorectal cancer (CRC) is one of the most frequent neoplasms and an important cause of morbidity in the developed world and it is increasingly frequent in developing countries. There has been increasing evidence from clinical trials that chemotherapy treatment greatly improves the chances of healing and survival in CRC patients with stages III or higher. 5-FU has been the cornerstone for first-line CRC systematic chemotherapy for many years and its combination with oxaliplatin (that is, FOLFOX) has become the most common regimen for CRC patients. However, the toxicities associated with the administration of these drugs have sometimes overshadowed the benefits they deliver. Patients treated with 5-FU commonly exhibit gastrointestinal and haematopoietic toxicities, whereas FOLFOX- treated patients are at risk of developing sensory neuropathy, which may endure even long after cessation of chemotherapy. All these side effects are thought to be mainly due to the narrow therapeutic indexes of most anticancer drugs.

Until recently, the investigation of the inheritance factors underlying the diverse response to CRC chemotherapy agents had mainly focused on candidate-gene studies, in which variants in genes coding for proteins involved unspecific pathways, such as drug absorption, metabolism or target molecules, were screened for evidence of their association with therapy outcome. For instance, variants in candidate genes such as DPYD, TYMS or UGT1A1 have already been linked to the development of ADRs in CRC patients treated with chemotherapy. However, these relatively rare and large-effect phenotypes might not apply to the majority of drugs or patients. It is expected that for common pharmacogenetic traits, as for most diseases, the inheritance patterns behind these responses are complex, with an interplay of multiple variants in the determination of the final outcome. In this sense, candidate-gene association studies have been proven to be an important tool for the identification of some of these other variants. However, the simultaneous study of higher numbers of variants has become increasingly necessary in order to evaluate the full contribution of inheritance to drug response.

Genome-Wide Association Study (GWAS) has proved to be an important tool for this purpose. The main advantage of this type of study in opposition to gene-based strategies is that they may be able to identify variants in genes or pathways that have not been implicated in mediating drug response so far. New SNPs related with ADRs and CNVs with GWAS approaches, some of them with high ORs putting bricks on the path to personalized medicine on CRC treatment.

This work has been supported by a grant from the Fondo de Investigación Sanitaria/FEDER (PS09/02368) and for the HiCi program at the Center of Excellence in Genomic Medicine Research, King Abdulaziz University, Kingdom of Saudi Arabia. Some of the results presented here have been obtained in the framework of the EPICOLON consortium.

